# Effects of *cis* and *trans* Genetic Ancestry on Gene Expression in African Americans

**DOI:** 10.1371/journal.pgen.1000294

**Published:** 2008-12-05

**Authors:** Alkes L. Price, Nick Patterson, Dustin C. Hancks, Simon Myers, David Reich, Vivian G. Cheung, Richard S. Spielman

**Affiliations:** 1 Department of Epidemiology, Harvard School of Public Health, Boston, Massachusetts, United States of America; 2 Department of Biostatistics, Harvard School of Public Health, Boston, Massachusetts, United States of America; 3 Program in Medical and Population Genetics, Broad Institute of MIT and Harvard, Cambridge, Massachusetts, United States of America; 4 Department of Genetics, University of Pennsylvania School of Medicine, Philadelphia, Pennsylvania, United States of America; 5 Department of Statistics, University of Oxford, Oxford, United Kingdom; 6 Department of Genetics, Harvard Medical School, Boston, Massachusetts, United States of America; 7 Department of Pediatrics, University of Pennsylvania School of Medicine, Pennsylvania, United States of America; 8 The Children's Hospital of Philadelphia, Philadelphia, Pennsylvania, United States of America; 9 Howard Hughes Medical Institute, Philadelphia, Pennsylvania, United States of America; The University of Queensland, Australia

## Abstract

Variation in gene expression is a fundamental aspect of human phenotypic variation. Several recent studies have analyzed gene expression levels in populations of different continental ancestry and reported population differences at a large number of genes. However, these differences could largely be due to non-genetic (e.g., environmental) effects. Here, we analyze gene expression levels in African American cell lines, which differ from previously analyzed cell lines in that individuals from this population inherit variable proportions of two continental ancestries. We first relate gene expression levels in individual African Americans to their genome-wide proportion of European ancestry. The results provide strong evidence of a genetic contribution to expression differences between European and African populations, validating previous findings. Second, we infer local ancestry (0, 1, or 2 European chromosomes) at each location in the genome and investigate the effects of ancestry proximal to the expressed gene (*cis*) versus ancestry elsewhere in the genome (*trans*). Both effects are highly significant, and we estimate that 12±3% of all heritable variation in human gene expression is due to *cis* variants.

## Author Summary

Variation in gene expression is a fundamental aspect of human phenotypic variation, and understanding how this variation is apportioned among human populations is an important aim. Previous studies have compared gene expression levels between distinct populations, but it is unclear whether the differences that were observed have a genetic or nongenetic basis. Admixed populations, such as African Americans, offer a solution to this problem because individuals vary in their proportion of European ancestry while the analysis of a single population minimizes nongenetic factors. Here, we show that differences in gene expression among African Americans of different ancestry proportions validate gene expression differences between European and African populations. Furthermore, by drawing a distinction between an African American individual's ancestry at the location of a gene whose expression is being analyzed (*cis*) versus at distal locations (*trans*), we can use ancestry effects to quantify the relative contributions of *cis* and *trans* regulation to human gene expression. We estimate that 12±3% of all heritable variation in human gene expression is due to *cis* variants.

## Introduction

Admixed populations are uniquely useful for analyzing the genetic contribution to phenotypic differences among humans. Phenotypic differences that are observed among human populations may have systematic non-genetic causes, such as differences in environment [1],[2]. However, in an admixed population such as African Americans, such differences are minimized and the only systematic differences among individuals are in the proportion of European ancestry, which can be accurately inferred using genetic data. Several recent epidemiological studies in African Americans have taken advantage of this, showing that many phenotypic traits vary with the proportion of European ancestry [3]–[5]. Here, we apply this idea to analyze population differences in gene expression.

Gene expression is a fundamental determinant of cellular phenotypes, and understanding how gene expression variation is apportioned among human populations is an important aspect of biomedical research, as has been true for apportionment of human genetic variation at the DNA level [6]. Recently, four studies analyzed lymphoblastoid cell lines from HapMap samples and reported that a large number of expressed genes exhibit significant differences in gene expression among continental populations [7]–[10]. However, results of these studies may be affected by non-genetic factors such as differences in environment, differences in preparation of cell lines, or batch effects [2],[9],[11],[12]. In particular, a recent review article has suggested that much of the expression variation across populations is caused by environmental factors [13]. On the other hand, analyses of expression differences that are correlated to ancestry within an admixed population are robust to all of these concerns.

In this study, we analyzed lymphoblastoid cell lines from 89 African-American samples and investigated the relationship between expression levels of ∼4,200 genes and the proportion of European ancestry. We compared the results with those predicted from the differences in expression levels between 60 European samples (CEU from the International HapMap Project) and 60 African samples (YRI from HapMap) [6]. We confirmed the existence of heritable gene expression differences between CEU and YRI by showing a highly significant correspondence between observed CEU vs. YRI differences (i.e. differences between sample means) and the expression differences predicted by ancestry differences among African Americans. Notably, the correspondence holds regardless of whether differences between CEU and YRI are large or small. This suggests that the effects of heritable population differences on variation in gene expression are widespread across genes, mirroring population differences at the DNA level [6].

Heritable variation in gene expression may be due to *cis* or *trans* variants. Previous studies in humans have been successful in mapping both *cis* and *trans* effects, but the results they provide are far from complete, due to limited sample sizes [14], [15], [9], [16]–[20]. In particular, the relative number of *cis* vs. *trans* associations that were reported varies widely across these studies, perhaps due to differences in power or choices of significance thresholds [13]. Thus, the overall extent of *cis* vs. *trans* regulatory variation in human gene expression has not yet been established. Here, by measuring how gene expression levels across all genes vary with local ancestry (0, 1 or 2 European chromosomes) either proximal to the expressed gene (*cis*) or elsewhere in the genome (*trans*), we estimate that 12±3% of heritable variation in human gene expression is due to *cis* variants.

## Materials and Methods

### Genotype Data

100 African-American (AA) samples from the Coriell HD100AA panel were genotyped on the Affymetrix SNP 6.0 GeneChip. Genotyping was conducted at the Coriell Genotyping and Microarray Center, and the genotype data was obtained from the NIGMS Human Genetic Cell Repository at Coriell (see Web Resources). In addition, genotype data from 60 European (CEU), 60 African (YRI), 45 Chinese (CHB) and 44 Japanese (JPT) samples was obtained from Phase 2 HapMap [6] (see Web Resources). We restricted all analyses to 595,964 autosomal markers with <5% missing data in AA samples and <5% missing data in Phase 2 HapMap samples, with A/T and C/G markers excluded so as to preclude any ambiguity in strand complementarity. Our analyses were not sensitive to the number of markers used. Two AA samples which we identified as cryptically related to other AA samples were excluded from the set of samples used for principal components analysis.

### Genome-Wide and Local Ancestry Estimates of AA Samples

Local ancestry (0, 1 or 2 European chromosomes) at each location in the genome was estimated for each AA sample using the HAPMIX program, a haplotype-based approach that has been shown to attain an *r* ^2^ of 0.98 between inferred local ancestry and true local ancestry in simulated African-American data sets (A.L.P., N.P., D.R. & S.M., unpublished data; see Web Resources, specifically http://www.stats.ox.ac.uk/˜myers/software.html). The HAPMIX program inputs AA genotype data and phased CEU and YRI data from Phase II HapMap [6], and outputs the estimated probability of 0, 1 or 2 European chromosomes at each location in the genome. The weighted sum of these probabilities (multiplied by 0.00, 0.50 or 1.00, respectively) forms an estimate of local % European ancestry. Genome-wide ancestry was computed as the average of estimated local ancestry throughout the genome.

### Gene Expression Data

Lymphoblastoid cell lines for 60 HapMap CEU, 60 HapMap YRI and the Coriell HD100AA samples were obtained from Coriell Cell Repositories (see Web Resources). Gene expression was assayed using the Affymetrix Genome Focus Array, as described previously [7]. We restricted our analysis to the 4,197 genes on the array that are expressed in lymphoblastoid cell lines [7]. The gene expression data is publicly available (GEO accession number GSE10824) (see Web Resources). For HD100AA samples, we excluded two cryptically related samples (see above), four samples identified as genetic outliers (see Results), and five samples for which gene expression measurements were not obtained, so that 89 AA samples were included in gene expression analyses.

### Validation Coefficient *c* of CEU versus YRI Gene Expression Differences in AA Samples

For each gene *g*, we normalized gene expression measurements for CEU and YRI to have mean 0 and variance 1 across 120 CEU+YRI samples, and normalized gene expression measurements for AA by applying the same normalization for consistency. We implicitly assume an additive genetic model in which gene expression has genetic and non-genetic components, with part of the genetic component predicted by ancestry. Let *e_gs_* denote normalized gene expression of gene *g* for sample (i.e. individual) *s*. Let *θ_s_* denote the genome-wide European ancestry proportion of sample *s*, so that *θ_s_* has value 1 for CEU samples and 0 for YRI samples as above, and fractional values for AA samples. We consider a model in which *e_gs_* = *a_g_θ_s_*+*ν_gs_* for CEU and YRI samples and *e_gs_* = *ca_g_θ_s_*+*ν_gs_* for AA samples, where *c* is a global parameter and *ν_gs_* represents the residual contribution to gene expression that is not predicted by ancestry. Thus, the parameter *c* represents a validation coefficient measuring the aggregate extent to which the observed gene expression differences *a_g_* between CEU and YRI (differences between sample means) are heritable.

We implemented two different approaches for fitting the parameters *c* and *a_g_* of this model: (1) Starting with the initial guess *c* = 1, we alternated computing maximum likelihood estimates for *a_g_* (for all *g*) conditional on *c*, and computing a maximum likelihood estimate for *c* conditional on *a_g_* (for all *g*), and iterated to convergence. In each case, the maximum likelihood estimates were obtained via linear regression (with a separate linear regression for each *g* when estimating *a_g_*, and a single linear regression when estimating *c*). (2) For each *g*, we estimated values *ã_g_*_,CEU+YRI_ by regressing *e_gs_* against *θ_s_* using CEU and YRI data only, and *ã_g_*_,AA_ by regressing *e_gs_* against *θ_s_* using AA data only. We then regressed *ã_g_*_,AA_ against *ã_g_*_,CEU+YRI_ to obtain an estimate of *c*. In this computation, we scaled our estimates of *ã_g_*_,CEU+YRI_ using the sampling error correction *ξ* (described below in Computation of *Q*_ST_) to remove the effect of sampling error on the denominator Σ*_g_*(*ã_g_*_,CEU+YRI_)^2^ of our estimate of *c*. (On the other hand, we note that sampling noise in the AA data does not bias our computation of *c*, whose expected value does not change when noise is added to *ã_g_*_,AA_). We observed that approaches (1) and (2) produced identical estimates of *c*, indicating that both approaches are effective in finding the best fit to the model. We followed approach (2) to plot *ã_g_*_,AA_ vs. *ã_g_*_,CEU+YRI_ and to compute estimates of *c* specific to different values of |*ã_g_*_,CEU+YRI_|.

### Validation Coefficient *c* using Genotype Data Instead of Gene Expression Data

We repeated the above computation using genotype data instead of gene expression data. We restricted the analysis to markers in which the average of CEU and YRI frequencies was between 0.05 and 0.95. Although AA genotypes at each marker were used twice in this computation—both for estimating genome-wide ancestry using all markers and for measuring the effect of genome-wide ancestry on genotype at a specific marker—we note that with hundreds of thousands of markers, our estimate of genome-wide ancestry is negligibly impacted by data from a specific marker.

### Validation Coefficients *c_cis_* and *c_trans_*

We investigated the effects of *cis* ancestry and *trans* ancestry on gene expression in AA. Roughly, we define *cis* ancestry as the local ancestry at the gene whose expression is being analyzed, and *trans* ancestry as the average ancestry at non-*cis* regions. We extended our above model by letting *e_gs_* = *c_cis_a_g_γ_gs_*+*c_trans_a_g_θ_s_+ν_gs_* for AA samples, where *γ_gs_* denotes the estimated local ancestry of sample *s* at the SNP closest to the center of gene *g* (*cis* locus; average of transcription start and transcription end positions). We note that although *trans* ancestry is theoretically defined as the average ancestry at non-*cis* regions, this quantity is in practice virtually identical to *θ_s_* because *cis* regions (regardless of the precise definition of *cis*) form an extremely small proportion of the genome. Because chromosomal segments of ancestry in AA typically span >10 Mb [21], it is nearly always the case that a gene lies completely within a single ancestry block, so that our analysis is not sensitive to the choice of genomic location used to define *cis* ancestry *γ_gs_*. The probabilistic estimates of local ancestry produced by HAPMIX are extremely accurate (see above), so that *γ_gs_* is typically close to 0.00, 0.50 or 1.00 (corresponding to 0, 1 or 2 copies of European ancestry). To avoid complications in local ancestry analyses on the X chromosome, we restricted this analysis to 4,015 autosomal genes. (Analyses involving global ancestry were not affected by inclusion or exclusion of genes on the X chromosome.) We estimated the global parameters *c_cis_* and *c_trans_* as above, accounting for the correlation between genome-wide and local ancestry by using residual values of *γ_gs_* (adjusted for *θ_s_*) to compute *ã_cis_*_,*g*,AA_ (and conversely for *ã_trans_*_,*g*,AA_).

### Computation of *Q*_ST_

Let *F* denote the proportion of total variance in gene expression that is attributable to population differences. For quantitative traits with an additive genetic basis, the quantity that is analogous to single-locus estimates of *F*_ST_ is not *F*, but rather *Q*_ST_ = *F*/(2−*F*) (reviewed in [22]). This is a consequence of the contributions of genetic variation on two distinct haploid chromosomes, magnifying the effect of population differences under an additive genetic model. We computed both *F* and *Q*_ST_. For each gene *g*, we normalized gene expression measurements for CEU and YRI to have mean 0 and variance 1 across 120 CEU+YRI samples. We defined the ancestry *θ_s_* of sample *s* to be 1 if *s* is a CEU sample, and 0 if *s* is a YRI sample. As above, we modeled normalized expression of gene *g* for sample *s* as *e_gs_* = *a_g_θ_s_*+*ν_gs_*. Equivalently, under this definition, *a_g_* is equal to the difference in normalized gene expression between CEU and YRI samples. We defined *F* to be the quantity such that the *true* value of *a_g_* has mean 0 and variance 2*F* across genes [23]. For a specific gene, *a_g_θ_s_* has variance 0.25*a_g_* ^2^ and *ν_gs_* has variance 1–0.25*a_g_* ^2^ across CEU+YRI samples (these variances have expected value 0.5*F* and 1–0.5*F*, respectively). Due to sampling error, the *observed* difference *ã_g_* in normalized gene expression between CEU and YRI samples (i.e. the coefficient obtained from a regression of *e_gs_* on *θ_s_*) has variance 2*F*+(1–0.5*F*)/30, where 1/30 is the sum of reciprocals of CEU and YRI sample sizes. We thus estimated mean *F* as (Var*_g_*(*ã_g_*) – 1/30)/(2 – 0.5/30). The ratio between mean *F* and Var*_g_*(*ã_g_*)/2 represents a sampling error correction that we call *ξ*. We estimated median *F* as the median value of *ã_g_* ^2^/2 times *ξ*. The value of *ξ* was 0.93, indicating that the sampling error correction had only a minor effect on these computations. To account for differences between CEU and YRI due to non-genetic factors, we adjusted *F* by multiplying it by *c*. (We note that the scaled population differences *ca_g_* have variance that is *c* ^2^ times the variance of *a_g_*, but explain only the proportion *c* of the true component of variance that is attributable to ancestry.) We then computed *Q*_ST_ = *F*/(2−*F*). We calculated the standard error of our estimate of *F* via jackknife, repeating the computation of *F* 120 times with one of the 120 CEU+YRI samples excluded in each computation, and estimating the standard error as the standard deviation of the 120 estimates times the square root of 120.

### Web Resources

- http://ccr.coriell.org (Coriell Cell Repositories)
- http://ccr.coriell.org/Sections/Collections/NIGMS/GenotypeCopyData.aspx?PgId564&coll=GM (The NIGMS Human Genetic Cell Repository at Coriell)
- http://www.hapmap.org (International HapMap Project)
- http://www.ncbi.nlm.nih.gov/geo (Gene Expression Omnibus)
- http://www.stats.ox.ac.uk/˜myers/software.html (HAPMIX program)

## Results

### Genetic Data Show that African Americans Are Accurately Modeled using CEU and YRI

**Figure 1:**
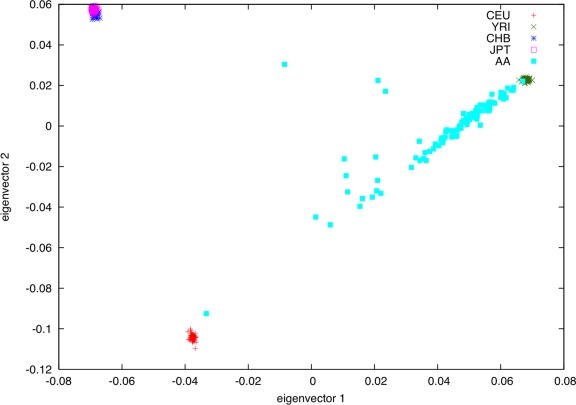
Principal components analysis of AA samples from Coriell together with HapMap samples. We display the top two principal components.

We analyzed Affymetrix 6.0 genotype data from the African-American panel of 100 samples from Coriell Cell Repositories, together with HapMap samples (see Materials and Methods). We first ran principal components analysis, using the EIGENSOFT software [24]. The top two principal components are displayed in Figure 1, in which most AA samples roughly lie on a straight line running from CEU to YRI (we excluded three genetic outliers with partial East Asian ancestry and one genetic outlier whose ancestry is very close to CEU from subsequent analyses). This suggests that the ancestry of the AA samples might be reasonably approximated as a mixture of varying amounts of CEU and YRI ancestry, as reported previously [21]. However, given the wide range of genetic diversity across Europe and particularly across Africa [23], we sought to test this hypothesis further. We removed related samples, genetic outliers, and samples without valid gene expression measurements to obtain a reduced set of 89 AA samples for subsequent analysis (see Materials and Methods). We computed *F*_ST_ values between the set of 89 AA samples and possible linear combinations *α*CEU+(1−*α*)YRI, adjusting for sample size. The lowest value of *F*_ST_ = 0.0009 was obtained at *α* = 0.21. Thus, the 89 AA samples are extremely well-modeled as a mix of CEU and YRI, with average ancestry proportions of 21% CEU and 79% YRI. Though this justifies our modeling approach using CEU and YRI, we caution against drawing historical inferences from this finding: because *F*_ST_ scales with the square of admixture proportion, it is possible that African Americans inherit a small percentage of their ancestry from a more diverse set of populations.

We estimated the genome-wide proportion of European ancestry for each the 89 AA samples (see Materials and Methods). Genome-wide ancestry proportions varied from 1% to 62% with a mean±SD of 21±14%; this ancestry distribution is similar to that in other AA data sets [21],[25]. Genome-wide ancestry estimates were strongly correlated (*r* ^2^>0.99) with coordinates along the top principal component (eigenvector with largest eigenvalue) (Figure 1).

### Gene Expression Levels Vary with Genome-Wide Ancestry in African Americans

We measured gene expression in lymphoblastoid cell lines from 60 CEU and 60 YRI samples from HapMap and 89 AA samples from Coriell, using the Affymetrix Genome Focus Array (see Materials and Methods). Our basic approach was to validate observed differences between CEU and YRI (differences between sample means) by analyzing the correlation between the genome-wide proportion of European ancestry estimated from SNP genotyping and the gene expression levels we measured in the AA cell lines. A caveat is that the proportion of European ancestry in African Americans might in principle be correlated to environmental variables. However, such correlations would not affect our approach unless they specifically tracked environmental differences between CEU and YRI. An additional caveat is that the Coriell panel of AA samples is known to be sampled from several (unknown) cities in the United States; AA samples from different U.S. cities might differ systematically in both the average proportion of European ancestry [21],[26] and in the preparation of cell lines. However, ancestry differences among AA populations in different U.S. cities are usually relatively small (standard deviation of 1% in Table 2 of [21]; standard deviation of 6% in Figure 2 of [26]), and in any case would not affect our approach unless differences in cell line preparation specifically tracked differences between CEU and YRI.

**Figure 2:**
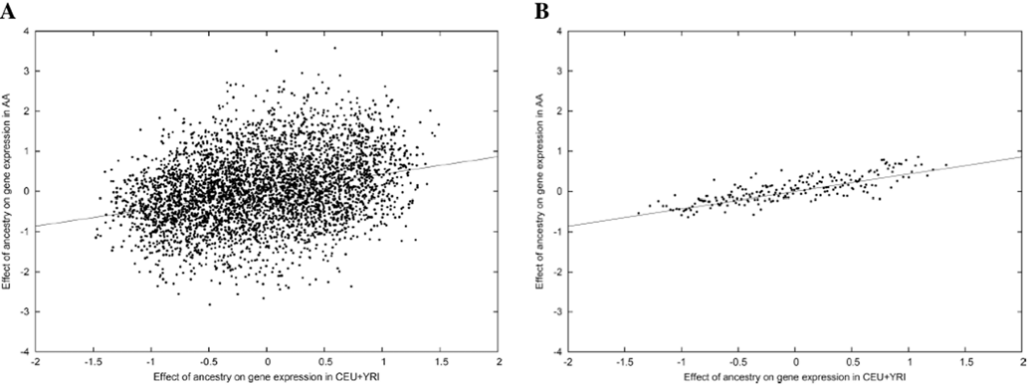
Gene expression differences between CEU and YRI are validated in AA samples. The y-axis shows the difference in normalized gene expression due to ancestry estimated from AA samples (*ã_g_*_,AA_) and the x-axis shows the difference in normalized gene expression due to ancestry estimated from CEU and YRI samples (*ã_g_*_,CEU+YRI_) (see Materials and Methods for details of normalization). (A) We plot each of the 4,197 genes separately. (B) For aid in visualization, the 4,197 genes are averaged into bins of 20 genes according to values of *ã_g_*_,CEU+YRI_; binning does not affect the slope of the plot. The slope of each plot is our estimate 0.43 of the parameter *c*.

Using the ancestry estimates and expression data at 4,197 genes for CEU, YRI and AA samples, we fit a model in which the effect of ancestry on gene expression at gene *g* is equal to *a_g_* per unit of European ancestry for CEU and YRI samples (so that *a_g_* is equal to the difference in mean expression level between CEU and YRI, which have ancestry 1 and 0 respectively), and equal to *ca_g_* per unit of European ancestry for AA samples, where *c* is constant across genes (see Materials and Methods). Thus, the global parameter *c* measures the extent to which observed gene expression differences between CEU and YRI are validated in AA, and therefore heritable. If systematic differences observed between CEU and YRI were entirely due to genetic factors, we would expect to see the same ancestry effects in AA samples, so that *c* = 1. On the other hand, under the hypothesis that observed differences between CEU and YRI are entirely due to *non*-genetic factors, we would expect *c* = 0. We note that our procedure for estimating *c* accounts for both experimental noise and sampling noise in the measurement of gene expression levels. Thus, assuming analogous normalizations for CEU, YRI and AA samples, our estimate of *c* is not dependent on the accuracy of our measurements; it is also independent of sampling effects.

**Figure 3:**
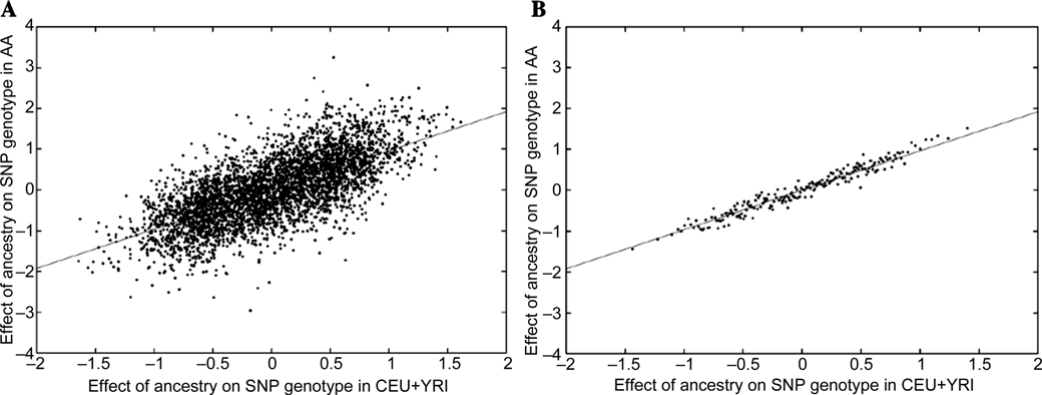
Genetic differences between CEU and YRI are validated in AA samples. Plots are analogous to Figure 2 except that genetic (SNP) data were used instead of gene expression data. (A) We plot a random subset of 4,197 markers, for visual comparison to Figure 2. (B) We average into bins of 20 markers. The slope of each plot is our estimate 0.96 of the parameter *c*.

Fitting the above model, we obtained *c* = 0.43, the slope of the regression line in Figure 2. With 4,197 genes analyzed, this estimate of *c* is different from zero with overwhelming statistical significance (P-value<10^−25^; 95% confidence interval [0.38,0.47]). Thus, gene expression differences among AA samples of varying ancestry strongly confirm that heritable differences contribute to observed gene expression differences between CEU and YRI. Performing the analogous computation with genotype data, we obtained *c* = 0.96, confirming that *c* is close to 1 for genetic effects (see Figure 3) and that modeling AA as a mix of CEU and YRI is appropriate for our analyses. The deviation between *c* = 0.96 and the expected value of 1 is discussed in Text S1.

We investigated whether the correspondence between observed CEU vs. YRI gene expression differences and expression differences due to ancestry among AA is concentrated in genes with large differences between CEU and YRI. If only a fraction of genes were truly differentiated, as suggested by previous studies, then genes with large observed CEU vs. YRI differences would be more likely to be truly differentiated and would show stronger validation in AA. For example, when we simulated a mixture model in which *c* = 0.43 for the set of all genes but only 50% of genes are truly differentiated between CEU and YRI, we obtained a larger value of *c* = 0.53 for genes in the top 10% of observed CEU vs. YRI differences (see Text S1). However, Figure 2 shows no evidence of nonlinear effects. Indeed, we recomputed *c* using only genes in the top 10% of the magnitude of observed CEU vs. YRI differences, and obtained *c* = 0.44, which is similar to the value of 0.43 using all genes. These results suggest that population differences in gene expression are not restricted to a fraction of genes but in fact are widespread across genes, mirroring population differences at the DNA level [6].

We considered whether the alternative approach of analyzing the AA data independently, without regard to differences between CEU and YRI, would be informative about differences in gene expression due to ancestry. We determined that the AA data analyzed separately contains too much sampling noise for that approach to be useful here (see Text S1). A related observation is that efforts to estimate the proportion of genes with population differences in gene expression, for example using the previously described [27] lower bound statistic 1–*π*_0_, may produce substantial underestimates in the case of data sets affected by sampling noise (see Text S1).

### Effects of *cis* versus *trans* Ancestry on Gene Expression in African Americans

The effect of ancestry on gene expression in African Americans may be due either to variation in regulatory variants proximal to the gene (*cis*) or to variants elsewhere in the genome (*trans*). We inferred the local ancestry of each AA sample at each location in the genome (see Materials and Methods). A description of how local ancestry varies across the genome (either across or within samples) is provided in Text S1. We quantified the extent to which the validation of CEU-YRI expression differences in AA was attributable to *cis* or *trans* effects in AA by computing validation coefficients *c_cis_* and *c_trans_* (see Materials and Methods). We obtained *c_cis_* = 0.05 and *c_trans_* = 0.38. As expected, the sum *c_cis_*+*c_trans_* is very close to the validation coefficient *c* that was obtained using genome-wide ancestry only (see Text S1). Both *c_cis_* (P-value = 6×10^−6^; 95% confidence interval [0.03,0.07]) and *c_trans_* (P-value<10^−25^; 95% confidence interval [0.33,0.43]) were significantly different from zero. Thus, only a small fraction of the effect of ancestry on gene expression is due to ancestry at the *cis* locus. On the other hand, performing the analogous computation with genotype data, we obtained *c_cis_* = 0.99 and *c_trans_* = –0.03, indicating as expected that the effect of ancestry on genotype is entirely due to ancestry at the *cis* locus, and confirming the high accuracy of our estimates of local ancestry.

We estimate the proportion *π_cis_* of heritable gene expression variation between Europeans and Africans that is due to *cis* variants as *c_cis_*/(*c_cis_*+*c_trans_*) = 12%, with a standard error of 3%. An important question is whether our estimate of *π_cis_* can be extended to *all* heritable variation in human gene expression. If the relative magnitude of *cis* vs. *trans* effects were different for all variation as compared to population variation—equivalently, if the relative magnitude of population variation relative to all variation were different for *cis* vs. *trans* effects—then the answer to this question would be no. To evaluate whether this is the case, we computed *F*_ST_(CEU,YRI) for ∼3,000 unique *cis* eQTL SNPs and ∼700 unique *trans* eQTL SNPs identified in a recent study of gene expression in human liver [20]. We obtained *F*_ST_ values of 0.158 for *cis* eQTLs and 0.154 for *trans* eQTLs, which were not significantly different from 0.159 for all HapMap SNPs (P-values = 0.79 and 0.51 respectively), based on standard errors computed using the EIGENSOFT software [6],[24]. Although this analysis involved eQTLs for liver tissue rather than lymphoblastoid cell lines, a reasonable assumption is that the same result holds for other tissue types. Thus, population variation does not appear to differ for *cis* vs. *trans* effects, implying that our estimate of *π_cis_* = 12±3% applies to all heritable variation in human gene expression.

### Proportion of Variation in Gene Expression Attributable to Population Differences

We estimated both the proportion of gene expression variation attributable to population differences, which we call *F*, and the quantity *Q*_ST_ = *F*/(2−*F*) which is analogous to *F*_ST_ for genetic (allele-frequency) data (see Materials and Methods). We obtained a mean *F* = 0.20 and median *F* = 0.12, similar to the median *F* = 0.15 from a previous analysis of CEU and YRI gene expression [8]. A jackknife calculation indicated that the standard error in our estimate of mean *F* was 0.02, corresponding to a 95% confidence interval of [0.15,0.25]. In our initial calculation of *F*, we ignored the possibility of non-genetic contributions to population differences. However, the fact that *c* is smaller than 1 implies that not all of the observed CEU vs. YRI differences are reflected in differences due to ancestry among AA. Some of these differences must reflect non-genetic factors. We therefore adjusted our estimates of *F* by multiplying them by *c* = 0.43 (see Materials and Methods). After this adjustment, we obtained a mean *F* = 0.09 and median *F* = 0.05. These estimates of *F* are substantially lower than those reported previously [8]. Our mean *F* corresponds to a *Q*_ST_ value of 0.05, which is lower than the *F*_ST_ of 0.16 that is observed in genetic data [6]. The lower value of *Q*_ST_ as compared to genetic data is unsurprising since *Q*_ST_ represents a proportion of total gene expression variation, which is expected to include both genetic and non-genetic components. We also note that if measurement variation is substantial, then the use of technical replicates to correct for the effects of measurement variation would lead to a higher value of *Q*_ST_.

## Discussion

We have shown how phenotypic variation in an admixed population can be coupled with variation in ancestry to shed light on differences between ancestral populations; our approach makes no assumptions about the population histories underlying the differences between the ancestral populations. We have applied this approach to gene expression in African Americans and shown that observed population differences (differences in sample means) between CEU and YRI in gene expression correspond, with overwhelming statistical significance, to differences among African Americans of varying ancestry, implying a substantial heritable component to the population differences. In reaching this conclusion via analysis of an admixed population, we eliminate confounding with non-genetic contributions to observed differences between the ancestral populations, which could result from differences in environment, differences in preparation of cell lines, or batch effects. The value of 0.43 for the “validation coefficient” *c* implies that both genetic and non-genetic effects contribute to observed population differences between CEU and YRI.

Interestingly, the validation coefficient *c* did not vary appreciably as a function of the magnitude of observed gene expression differences between CEU and YRI. This suggests that the effects of ancestry on gene expression are widespread across genes, as opposed to affecting only a fraction of genes. Although there exist genes for which the observed effect of ancestry on expression levels is close to zero (Figure 2), this does not rule out small ancestry effects at these genes, as similar results are observed in genetic data (Figure 3) in which it is commonly believed that ancestry affects 100% of common SNP frequencies. Indeed, if ancestry affects genotype and genotype affects gene expression­ (as indicated by previous studies reporting a substantial heritable component to gene expression [16],[17]), then the presence of ancestry differences at almost all expressed genes seems a not unreasonable hypothesis, and one with which our results are entirely consistent. However, just as with DNA variation, it is clear that population differences in gene expression represent only a small fraction of the overall variance, most of which is due to variation within populations.

In addition to validating the aggregate effects of ancestry on human gene expression, we were able to partition heritable variation into *cis* and *trans* effects, which would not be possible in a simple comparison of continental populations. Our admixture approach was fruitful despite the small magnitude of differences between human subpopulations. Our distinction between *cis* and *trans* effects is somewhat imprecise, due to the extended length (>10 Mb) of segments of continental ancestry in African Americans, but this has little effect on our conclusions, since a 10 Mb region represents a proportion of the genome that is much smaller than the 12% proportion of heritable variation in gene expression that we attribute to variation at the *cis* locus. Comparing our results to results obtained in other species, we note that two recent studies of gene expression in *Drosophila* also reported that *cis* effects explain a small fraction of heritable variation [28],[29], although previous *Drosophila* studies had suggested a larger role for *cis* effects [30],[31]. Our results have broad ramifications for future efforts to map the genetic regulation of gene expression. However, conclusions drawn from gene expression measured in lymphoblastoid cell lines do not necessarily extend to other tissue types, motivating further investigation. Going forward, admixed populations will continue to be useful for understanding and mapping gene expression and other phenotypes.

## Supporting Information

Text S1 Supplementary Note.
(0.02 MB PDF)
