# Supplementary material for: Effects of cis and trans Genetic Ancestry on Gene Expression in African Americans
Source: PLoS Genet. 2008 Dec 5;4(12):e1000294. doi: 10.1371/journal.pgen.1000294 (PMC2586034; doi:10.1371/journal.pgen.1000294)
Supplement: Text S1 — Supplementary Note. (0.02 MB PDF) [file pgen.1000294.s001.pdf]

Supplementary Note

Deviation between *c* = 0.96 for genetic data and the expected value of 1. The value of c = 0.96 that we obtained from genetic data is significantly different from the expected value of 1 (95% confidence interval: [0.954,0.960]) that would be anticipated if AA were perfectly modeled as a linear combination of CEU and YRI. However, we determined that this linear combination is an imperfect approximation (F_{ST} = 0.0009; see Results). Although the F_{ST} value of 0.0009 is extremely small relative to F_{ST}(CEU,YRI) = 0.16, it is not insignificant relative to the proportion of genetic variation in AA that is attributable to genome-wide ancestry, which is equal to $(0.16)(0.14/0.50)^2 = 0.0125$ (since the standard deviation of genome-wide ancestry in AA is 0.14 as compared to 0.50 for CEU+YRI).

Mixture model simulations. To compare theoretical predictions to our actual data, we simulated a mixture model in which an underlying variable x is drawn from a mixture of two distributions and a noise variable y is subsequently added to produce an observed variable $x+y$. Intuitively, $x+y$ corresponds to differences observed in CEU vs. YRI, and x corresponds to heritable differences that are validated in AA. Let N(0,V) denote a normal distribution with mean 0 and variance V. We define $x \sim N(0,c/p)$ with probability p or x is identically zero with probability $1–p$, and define $y \sim N(0,1-c)$. Under this model, the coefficient for $x+y$ predicting x (or x plus independent noise) is equal to c. Setting $p = 0.50$ and $c = 0.43$, we estimated the regression coefficient for $x+y$ predicting x when restricting to the top 10% of values of $|x+y|$.

Limited sample size of AA data precludes analysis excluding CEU and YRI

We determined that, due to limited sample size and the relatively low variability in genome-wide ancestry among AA individuals (standard deviation of 14% as compared to 50% for CEU+YRI; note that effective sample size scales with the square of this quantity), the AA data contains too much sampling noise for an analysis excluding CEU and YRI to be useful. As a demonstration of this, we computed the previously described statistic $1-\pi_0$, which estimates the proportion of causal data points from an observed distribution of P-values, to try to infer the fraction of common SNPs whose frequency varies with continental ancestry based on genotype data from the 89 AA samples. Based on standard F_{ST}-based models, it is commonly believed that 100% of common SNP frequencies vary with continental ancestry to at least a small extent. However, based on genotype data from the 89 AA samples, which strongly replicate genetic differences between CEU and YRI ($c = 0.96$ above), the value of $1-\pi_0$ was equal to 28%, which is much lower than 100%. The statistic $1-\pi_0$ represents a lower bound which has proven useful in a variety of contexts, but our analysis shows that this lower bound may not be very informative in data sets of limited sample size, in which causal data points may have P-values that are not statistically significant. This observation also applies to the use of this statistic, or other lower bounds, to estimate the proportion of genes with population differences in gene expression. On the other hand, our validation analyses which analyze AA data in conjunction with CEU and YRI data are not affected by the limited sample size of the AA data (see Materials and Methods).

Variation in local ancestry across the genome

We computed local ancestry estimates γ_{gs} for sample s at gene g as described in the main text. The mean ± SD of γ_{gs} across samples s and genes g was 21 ± 29%. The standard deviation

matched the theoretical expectation for a sample with 21% genome-wide ancestry which has 2 European copies with probability $(0.21)^2$, 1 European copy with probability 2(0.21)(0.79) or 0 European copies with probability $(0.79)^2$. We also computed the average ancestry γ_g (i.e. the average across samples s of γ_{gs}) for each gene g. The mean ± SD of γ_g across genes g was 21 ± 3%, as expected under a binomial model (variance equal to $(0.21\times 0.79)/(2\times 89)$). Values of γ_g ranged from 13% to 31%, but these deviations from the mean of 21% were not statistically significant after applying a Bonferroni correction (either for 4,015 genes tested, or for hundreds of independent loci based on ancestry block sizes on the order of 10Mb).

Relationship between c, c_{cis} and c_{trans}. The variation in local (*cis*) ancestry γ_{gs} (standard deviation = 29%; see above) is considerably larger than the variation in genome-wide (*trans*) ancestry θ_s (standard deviation = 14%; see Results). In fact, fixing g and letting s vary, we can view γ_{gs} as binomially sampled from θ_s, i.e. γ_{gs} is equal to θ_s plus sampling noise. We confirmed this by computing for each g the regression coefficient (across samples s) for θ_s for predicting γ_{gs}. The average value of this regression coefficient (averaging across g) was equal to 0.97. Under the assumption that γ_{gs} is equal to θ_s plus sampling noise σ_{gs}, and that the magnitude of $c_{cis}a_g\sigma_{\mathrm{gs}}$ is small relative to the overall noise variance v_{gs} of gene expression level e_{gs}, we would expect $c \approx c_{cis} + c_{trans}$, since $e_{gs} = c_{cis}a_g\gamma_{gs} + c_{trans}a_g\theta_s + v_{gs} = c_{cis}a_g(\theta_s + \sigma_{\mathrm{gs}}) + c_{trans}a_g\theta_s + v_{gs} \approx (c_{cis} + c_{trans})a_g\theta_s + v_{gs}$.
